# Supporting the psychosocial well-being of patients with Long COVID: patient-informed strategies from a qualitative study

**DOI:** 10.3389/fpsyt.2026.1837359

**Published:** 2026-09-23

**Authors:** Bryce D. Martinez, Jonathan G. Shaw, Steven M. Asch, Cassandra L. Bragdon, Amogha M. Paleru, Brianna Brasko, Anna Sophia Lessios, Tom C. Quach, Sindhushree Hosakote, Shelby Scott Lazarow, Samantha M. R. Kling, Marcy Winget, Linda N. Geng, Hector Bonilla, Cati Brown-Johnson

**Affiliations:** 1 School of Medicine, Stanford University, Stanford, CA, United States; 2 Evaluation Sciences Unit, Stanford School of Medicine, Palo Alto, CA, United States

**Keywords:** chronic conditions, disability, health services research, Long COVID, patient experience, post-viral condition, primary care, well-being

## Abstract

**Background:**

Long COVID, an illness characterized by long-lasting symptoms following SARS-CoV-2 infection, exacts a substantial toll on psychosocial well-being and overall health. Persistent physiological symptoms and associated experiences can negatively impact employment, daily functioning, identity, and social relationships. However, there is a gap in the literature on patient-derived recommendations for addressing the psychosocial impacts of living with Long COVID.

**Objectives:**

This study aimed to identify factors shaping the psychosocial well-being of individuals with Long COVID and elicit patient-generated strategies for addressing them.

**Methods:**

We conducted semi-structured individual interviews and Community Advisory Team (CAT) focus groups between November 2024 and June 2025 with 19 individuals who self-reported experience with Long COVID. Interviews and focus group meetings were recorded, transcribed, and qualitatively analyzed to identify factors influencing psychosocial well-being and participant-generated strategies for addressing them.

**Results:**

Three primary factors negatively influencing patient psychosocial well-being were identified: *identity disruption*, *isolation*, and *uncertainty*. Identity disruption was described as a combination of shifting capabilities and a loss of self-reliance. Isolation centered around reduced social participation and experiences being dismissed, doubted, or misunderstood. Uncertainty focused on the illness and treatment pathways, navigation of healthcare and disability systems, fluctuating symptoms, and an uncertain life trajectory. Participants identified strategies including case management, structured peer support, Long COVID awareness campaigns, and personal practices supporting adaptation and acceptance.

**Conclusion:**

Effectively treating and supporting Long COVID patients requires consideration of their broader state of psychosocial well-being and how it is influenced by identity disruption, isolation, and uncertainty. The impact of interventions targeting these specific factors should be studied to guide future intervention implementation.

## 1 Introduction

Long COVID, an illness characterized by long-lasting symptoms following COVID-19 infection, exacts a substantial toll on psychosocial well-being, including emotional, psychological, and social well-being, as well as physical and neuropsychiatric health (1–6). Long COVID has been associated with anxiety, depression, insomnia, post-traumatic stress disorder, and cognitive impairment (2, 7). Among U.S. adults, Long COVID may be responsible for 2.6 million cases of depression, 5.4 million cases of anxiety, and 1.9 million cases of individuals experiencing suicidal ideation, as estimated by Goodman et al. using symptom prevalence from a random community-based sample that was then extrapolated to the broader US adult population via contemporary CDC estimates of Long COVID prevalence (4). These mental health challenges likely arise from a complex interplay of factors, including aggravation of pre-existing conditions, lasting physiological symptoms, and grief over lost health and functional ability (8). The relationship between Long COVID and mental health may also be bidirectional, with studies suggesting that pre-existing psychological distress is associated with increased risk of self-reported post-COVID-19 conditions and diagnosed Long COVID (9, 10).

In addition to directly impacted health and well-being, many patients with Long COVID encounter fragmented healthcare systems and a lack of Long COVID-specific services, leaving them feeling dismissed or stigmatized by physicians unprepared to address the condition’s complexity (11). Additionally, social and financial stressors can erode patient well-being outside of clinical settings: prolonged illness may strain relationships and reduce social support while simultaneously limiting employment capabilities and creating negative financial impacts (5), contributing to heightened anxiety and depression (12).

A growing body of qualitative research has documented the psychosocial experiences of individuals with Long COVID (8, 13–22). However, comparatively less attention has focused on understanding what individuals with Long COVID identify as useful strategies for addressing these psychosocial challenges. A 2024 systematic review by Eberhardt et al. synthesized psychosocial themes as centering around debilitation, uncertainty, sources of support, meaning making: adjusting to a new normal, and experiences with healthcare, validated by studies in the UK, the Netherlands, Canada, and other countries (12–17, 23). U.S. experiences of Long COVID may differ on certain fronts due to unique barriers in health care access, social service systems, and broader social context (24). Studies conducted in Spanish-speaking countries also yield similar findings supporting a demonstrable psychosocial impact of Long COVID, with symptom burden, stigma, lack of recognition, and fragmented care or support being tied to experiences of distress and loneliness, but infrequently reflect patient voices (25–27). Eliciting patient-generated strategies may provide an important foundation for developing supportive interventions that are responsive to patients’ lived experiences and priorities.

To address this gap in the literature, we conducted a qualitative study of individuals with Long COVID in Northern California (USA) to examine factors influencing psychosocial well-being and elicit patient-generated ideas for supportive interventions. We further considered these recommendations alongside existing evidence in chronic health literature. This patient-centered approach aims to inform more responsive care and support strategies for the growing Long COVID population.

## 2 Methods

We conducted semi-structured interviews and Community Advisory Team (CAT) focus groups with a total of *n* = 19 individuals who self-reported patient experience with Long COVID. Interviews and focus group meetings were recorded, professionally transcribed (28), and analyzed using qualitative analysis software (29).

### 2.1 Sampling and participants

Participants were recruited via referrals from the Stanford Health Care Post-Acute COVID-19 Syndrome (PASC) Clinic and advertising at local community health fairs. This study utilized convenience sampling (30), with eligibility being self-reported experience with Long COVID as a patient. This criterion was intended to ensure that barriers to obtaining a formal diagnosis did not preclude participation. Individuals who declined or were otherwise unable to participate in the focus group sessions were offered the opportunity to participate in a single, one-on-one interview. Focus group members who had been unable to attend two consecutive sessions were also offered an individual interview to discuss topics from the missed focus group sessions. A total of 19 unique individuals participated in the study, with *n =* 7 participating in multiple focus group meetings, *n* = 14 participating in one-time individual interviews, and two individuals participating in both modalities.

### 2.2 Study context

This work was part of the Long COVID Care Resources and Education to Advance Community Health (REACH) program, funded by the Agency for Healthcare Research and Quality (AHRQ). The REACH program is focused on expanding and improving Long COVID care, including in underserved communities and for patients seen at Federally Qualified Health Centers (FQHCs). Within the REACH program, interviews and focus groups were conducted between November 2024 and June 2025 to gather healthcare-related experiences of Long COVID patients to inform clinical interventions. This analysis focuses on psychosocial well-being and was an embedded piece of our overall research and evaluation.

### 2.3 Data collection

#### 2.3.1 Focus groups: Long COVID patient community advisory team sessions

Five focus group sessions, with a total of *n* = 7 unique participants, were facilitated by the research team on Zoom in English. To reduce participation burden for individuals managing the fluctuating symptoms of Long COVID, attendance was intentionally flexible. Consequently, the subset of participants contributing to each session varied. Session attendance ranged from three to six participants, with an average attendance of 4.6 participants per session. On average, each participant attended 3.3 of the five CAT sessions. Sessions were 60 minutes long, occurred approximately one month apart, and followed a semi-structured discussion guide. Due to facilitation constraints, eligibility was limited to those with proficiency in English who also consented to being recorded.

Each of the five sessions focused on a distinct topic, with the following covered: Introductions, Clinician Education, Referral Pathways, Non-Medical Care Needs for Patients and Caregivers, and Patient Education. Participants received a $75 gift card per session attended as a thank-you for participating.

Data were collected between November 2024 and June 2025.

#### 2.3.2 Individual interviews

One-on-one interviews with *n* = 14 participants were conducted via Zoom or in person. Interviews followed a semi-structured guide, ranged from 30 to 60 minutes, and were audio-recorded using encrypted devices. Of the 14 individual interviews, seven were conducted in Spanish and seven in English. Two interviewees declined to be recorded and detailed notes were taken by the interviewer.

The semi-structured interviews explored topics including COVID-19 Care; Referral Experience; Needs, Support, and Caregivers; Patient Identity and Self-Perception; Community Engagement and Education; and Experience with Bias in Medicine. Example prompts from the interview guide can be found in **Table 1**, with the complete semi-structured interview guide provided in the **Supplementary Materials**. Interviewees received a $25 gift card as a thank-you for participating in an individual interview.

**Table 1:** Selected individual interview guide topics and example questions.

Topic	Question	Additional probe
Needs, Support, and Caregivers	*What is your experience with caregivers? This could be anyone that has helped you (a parent, a friend, a volunteer, etc.)*	*What would you say caregivers need to know when it comes to providing care for someone with Long COVID?*
Patient Identity and Self-Perception	*Can you tell me how living with Long COVID has affected how you see yourself or your role in your life—such as in your family, work, or community?*	*How has your sense of what you're capable of—physically, emotionally, or mentally—changed, if at all?*
Community Engagement and Education	*How would you describe your community’s awareness of Long COVID? (family, neighborhood, state, faith organization, etc.)*	*What do you think would be the best way to raise awareness and share information about Long COVID, and health more generally, in your community?*

Data were collected between November 2024 and June 2025.

### 2.4 Data analysis

Audio transcripts were generated and anonymized via TranscribeMe!, an external transcription service that meets HIPAA encryption and confidentiality standards (28). For Spanish-language interviews, a transcription by TranscribeMe! was completed in Spanish, then translated into English by TranscribeMe!, and the resulting English transcript was used for subsequent coding. The anonymized transcripts were then uploaded into NVivo 14 software for coding and analysis (29). All participant quotations presented in this manuscript were drawn directly from audio-recorded and transcribed study sessions rather than from field notes. Focus group and individual interview data were analyzed jointly as a single qualitative corpus and were given equal analytical weight during coding and thematic analysis. A preliminary codebook was deductively built with input from the research team, with six consensus coding meetings between study authors (BM, CB, and AP) used to discuss and revise code definitions and applications. Differences in coding were resolved through discussion during these six consensus coding meetings (31). An additional inductive coding process was applied to allow for the emergence of additional themes, including the subthemes relating to psychosocial well-being that are the focus of this study.

Throughout our thematic analysis, we aimed to identify participant-generated recommendations for addressing the challenges related to each theme. We then cross-referenced these participant recommendations with existing literature to identify conceptually aligned interventions, allowing for domain-specific recommendations within established frameworks of psychosocial well-being.

While our interviews were not bound to a single theoretical framework, our interpretation drew on the Stanford WELL framework and Maslow’s Hierarchy of Needs to situate findings within relevant models of psychosocial well-being (32–34). Using these two models during the interpretive phase helped us address limitations in clinical diagnosis and care; these broader models of well-being contextualized patient experiences beyond diagnoses. Maslow’s Hierarchy of Needs, a foundational framework of well-being, consists of five levels of needs: *physiological needs*, *safety needs*, *love and belonging*, *esteem*, and *self-actualization* (32, 35). While now over 70 years old, Maslow’s Hierarchy of Needs is consistently cited and adapted in modern research on well-being (36, 37). A more contemporary model of well-being, The Stanford WELL for Life framework (WELL), identifies eight key domains of well-being: *sense of self*, *positive emotions*, *social connectedness*, *resilience*, *negative emotions and experiences*, *sense of purpose and personal growth*, *physical health*, and *loneliness* (33, 34). The WELL framework is particularly valuable because of its development using open-ended data collection from a global longitudinal cohort of 31,625 participants spanning five countries including the USA, China, Taiwan, Singapore, and Thailand. This strengthens the cross-cultural and modern applicability of the framework (33, 34).

### 2.5 Researcher characteristics

The lead researcher (BM) for this study was a graduate student in Community Health and Prevention Research at Stanford, with an undergraduate background in Human Biology. The full research team (BM, JS, SA, CB, AP, BB, ASL, TQ, SH, SMRK, MW, LG, HB, SSL, CBJ) includes expertise in qualitative methods, patient engagement, Long COVID identification and treatment, and community primary care. The research team consists of women and men; undergraduate and graduate trainees in community health and medicine; degrees including MS, MA, MD, MPH, and PhD; and English and Spanish speakers.

### 2.6 Ethical considerations

The study protocol was approved by the Stanford University Institutional Review Board (Protocol #71861). All participants were reminded they could withdraw at any time without consequence. Clinicians were available to support a warm handoff in the event that a physical or mental health crisis occurred during data collection (this option was not needed). Additionally, following conversations, we shared information on free and low-cost resources with participants to support their health and well-being. Data were de-identified and stored securely on Stanford’s encrypted platform.

To protect the anonymity of our participants, qualitative data are not published here in full, but are available upon reasonable request. Publishing full transcripts/full qualitative data is not the standard in the field (38), particularly in the current technological environment where the potential ability to identify participating individuals based on their language choices, for instance through large language AI models, is a viable concern as explored in a recent preprint examining LLM-based re-identification (39).

## 3 Results

### 3.1 Factors influencing the psychosocial well-being of individuals with Long COVID

The primary factors patients identified as negatively influencing their psychosocial well-being included *identity disruption*, *isolation*, and *uncertainty* (**Table 2**). Identity disruption was described as a combination of shifting capabilities and a loss of self-reliance, such as depending on a partner to provide the sole income for a household. Physical and social isolation centered around reduced community interactions largely due to fatigue, and emotional isolation grew from repeatedly having Long COVID experiences dismissed, doubted, or misunderstood due to a lack of awareness about the illness. Lastly, a sense of overarching uncertainty was demonstrated by numerous questions about Long COVID’s biological underpinnings, treatment options, and disease progression. These questions, paired with fluctuating symptoms and capabilities, defined a widespread sense of an uncertain life trajectory.

**Table 2:** Factors influencing psychosocial well-being.

Factor	Explanation	Exemplar quotes
Disruption of Personal Identity	Long COVID symptoms undermined patients’ sense of self, particularly through loss of roles and activities central to their identity. Patients reported increased dependence on others, which conflicted with their previously held values of independence and self-sufficiency.	“I used to be a gym-going person, very outgoing before COVID. I've significantly gained a lot of weight due to my inability of being active with fitness and such.” (CAT 3) “I had always been a top salesperson at the company for five years, but I just couldn't keep up anymore … I would go to bed and hope that I never wake up again because that’s just how exhausted my body was.” (CAT 3) “The hardest thing for me has been that, before I got COVID, I was a very active woman … I didn’t depend on anyone … and now I can’t fend for myself because I’m too afraid to drive, to go out and everything.” (Interview 8)
Experiences of Isolation	Patients described physical and emotional isolation resulting from symptom burden, unpredictability, and misunderstanding driven by the invisibility of illness. Many withdrew from social spaces and reduced contact with friends.	“I’ve always been social, and I have become a complete recluse because I don’t know how to explain what I’m going through to people. And I tried once to one of my very best friends, and she didn’t understand.” (CAT 2) “I've dealt with friends and family saying, ‘Get over it already … You are fine. Nothing is wrong with you. You just got lazy.’ No one will ever know or truly realize that both mental and physical stressors are your true enemy now.” (CAT 2) “And I've become a hermit, pretty much - I'm getting a little better - because I don't trust other people with their germs … So it really limits me socially, to be around people.” (Interview 13)
Uncertainty	Participants reported distress from the unknown trajectory of their illness, unclear diagnosis, and conflicting medical guidance. Many feared that they may never fully recover. This ambiguity made long-term planning difficult and increased anxiety about work, health, and future roles.	“I'm still really struggling with long COVID … The therapist I was talking to said I might have to accept that this is my new life. That part’s really difficult because I’m hoping that I’ll eventually be who I was again. And I’m not sure that I will.” (CAT 2) “[The Long COVID specialist] mentioned that I could be on permanent disability if I wanted to, but [the other doctor] says no. He's not going to sign anything … He says I'm too young. Yes, I am young, but he doesn't know what I’m feeling. Only I know what I feel." (Interview 8) “Why do some people heal with COVID and some people don't? I just don't know … I started doing craniosacral. I was starting counterstrain, acupuncture … What else have I done? Massage. I soak my feet in something called fulvic minerals.” (Interview 2)

### 3.2 Factor 1: disruption of personal identity

Long COVID symptoms impacted patients’ abilities to engage in core activities, roles, and former capabilities, undermining their sense of personal identity. Many drew a clear line between life before and after this illness. This change brought grief, distress, and, for some, existential crises.

#### 3.2.1 Shifting capabilities and roles

Patients described losing the ability to perform activities and fulfill roles central to their identity. This included feeling less capable of physical activity, caring for children, excelling at work, and keeping up with academic responsibilities.

One participant described the dramatic transformation of their capabilities from being able to manage days full of yard work, errands, and supporting elderly family members to days of extreme exhaustion and pain.

“By 6:00 PM, I already cannot ignore my body screaming to shut down. Energy is beyond spent. Muscular pain is intense. Breathing is labored. Sitting and standing is a massive concern because you feel like you will collapse. Legs and arms are heavy. You cannot push through it anymore … So you tread lightly.” (Community Advisory Team Meeting #2)

Others, though less severely limited, described the emotional toll of losing quality time with family. One parent described missing formative experiences with their children.

“[My family has] lost a lot of quality time, camping, hiking—we’re very outdoorsy type of people—so [we’re] trying to adapt with what I can do as best to my abilities.” (Community Advisory Team Meeting #2)

Even moderate symptoms of brain fog and fatigue were barriers to daily functioning. Activities that were once routine, like attending classes, going to the movies, or doing household chores, now required significant effort or were outright impossible. Job loss due to illness-related performance issues was common, and some participants feared returning to work because of unpredictable symptoms and limited accommodations.

“And then I got let go from work for performance. I had always been a top salesperson at the company for five years, but I just couldn’t keep up anymore. I was super exhausted every single day to the point that—I’m not a suicidal person, but I would go to bed and hope that I never wake up again because that’s just how exhausted my body was.” (Community Advisory Team Meeting #3)

For many, the result was a painful feeling of being a diminished version of their former selves. The inability to meet personal or external expectations deepened this sense of identity loss.

#### 3.2.2 Loss of self-reliance and control

Patients described feeling less capable of managing daily responsibilities and more dependent on those around them, highlighting a loss of self-reliance and control. For those who saw independence as core to their identity, this change brought guilt, frustration, and fears of burdening family members.

One participant, who had previously juggled motherhood and extended workweeks, captured this shift and the pain it evoked:

“The hardest thing for me has been that, before I got COVID, I was a very active woman. I could work for up to 11 days in a row without rest, even though I have three children … I did everything, I didn’t depend on anyone. I always fended for myself and now I can’t fend for myself because I’m too afraid to drive, to go out.” (Interview 8)

Rebuilding even small forms of control, like managing a short outing alone, became milestones on the long path to recovery.

“I could not get close to people because I was afraid of them. Now I can be around people, socialize a little more, but before, no. I didn’t want to get close to anyone. And I didn’t want anyone to get close to me either. I go to the store by myself … I know and hope that little by little I am going to improve my life. It has been very difficult, but nothing is impossible.”(Interview 8)

### 3.3 Factor 2: experiences of isolation

A pervasive sense of isolation shaped experiences of living with Long COVID. Symptom burden and fears of reinfection reduced social contact, driving physical and social isolation. Simultaneously, the lack of a broadly shared understanding of the condition fostered emotional isolation, with participants feeling alienated and disconnected.

#### 3.3.1 Physical and social isolation

Fatigue and cognitive impairment limited participation in social activities, with many participants stating that they lacked the stamina for sustained interaction. Previously fulfilling social lives became severely curtailed by Long COVID, with some participants describing a complete social withdrawal and subsequent loneliness.

“When we’re going through these slumps with no energy, it’s almost hard to do really anything and have conversations with people. So that has prevented me a lot of the time from having a lot of progress with getting through it all just because it takes so much time and energy.” (Community Advisory Team Meeting #1)

Participants also shared how overwhelming symptoms created unpredictable “crashes” which made even digital forms of connection feel impossible.

“I go through these immense crashes where I literally cannot talk to anybody … I can’t communicate through text, through email. I mean, this is quite ridiculous, but just to share how many texts are on my cell phone that I have not even answered are 347.” (Community Advisory Team Meeting #2)

Beyond the direct impact of symptoms, concerns about reinfection frequently came up as a reason for avoiding public outings or socializing.

“And I’ve become a hermit, pretty much … because I don’t trust other people with their germs … So it really limits me socially, to be around people … Let’s say [my family] want[s] to go to a concert. I’m not going to go because, I mean, I can’t control it.” (Interview 13)

#### 3.3.2 Emotional isolation: facing a lack of awareness and understanding

A second dimension of isolation stemmed from the emotional toll of feeling unseen and misunderstood. While symptoms could be debilitating, they were usually invisible. This drove assumptions that patients were exaggerating or imagining their illness, creating a disconnect between their lived experience and the perceptions of those around them. The lack of recognition brought frustration and emotional withdrawal.

“I’ve dealt with friends and family saying, ‘Get over it already. It’s not a real condition … Nothing is wrong with you. You just got lazy.’ No one will ever know or truly realize that both mental and physical stressors are your true enemy now.” (Community Advisory Team Meeting #2)

Participants described difficulty communicating their experience with members of their support networks, even when those individuals were well intentioned. In an effort to conserve energy and avoid instances of invalidation, participants often stopped trying to explain their experience altogether.

“I’ve always been social, and I have become a complete recluse because I don’t know how to explain what I’m going through to people. And I tried once to one of my very best friends, and she didn’t understand.…I just realized unless somebody’s going through it, it’s just not really worth my energy to try and explain it to somebody … it’s just easier for me to just disengage.” (Community Advisory Team Meeting #2)

Considering these challenges, peer validation was seen as a vital mitigating force. Participants reported that support groups, usually online, offered recognition and a shared identity not found elsewhere, while offering an opportunity to support other Long COVID patients and organize collective advocacy efforts. The focus group sessions themselves were commonly cited as one of the few places where participants felt fully seen and heard (**Table 2**).

Participants reported wishing for greater efforts to spread information about, and raise awareness of, Long COVID. They emphasized the reach of social media and viewed it as an effective platform for disseminating information widely within their communities. This was seen as a foundational step toward increased understanding and emotional connection (**Table 3**).

**Table 3:** Participant-generated mitigation strategies: exemplar quote evidence supporting each mitigation strategy.

Factor	Participant-generated mitigation strategies & quotes
Disruption of Personal Identity	Home-based Services and Assistive Technologies “I have some random notebooks here and there, and they're all spread out, and I lose everything. And I just need something to collaborate what I'm experiencing that tracks it… I think that might be helpful for people to have something that they can maybe electronically journal that saves it.” (CAT 3) Flexible Employment Opportunities (e.g., Part-time Roles, Remote Work) “Are there communities …that support people that have long COVID, like job resources, where when they have an on day, they can do some work, and when they have an off-day, part-time kind of work?” (CAT 1) Therapy and Clinical Mental Health Support “I'm looking into different avenues to help manage the stress and anxiety…people need to recognize that long COVID probably created some type of major depressive disorder and anxiety, which it did for me. And that would be the first step is needing to get help in that.” (CAT 4) Cultivation of Gratitude and Positivity “I'm trying … spiritual reinforcement, be a little bit more positive of how things are in perspective…"Hey, we're lucky to be here on a day-to-day basis," and just really support each other and give each other the encouragement.” (CAT 2)
Experiences of Isolation	Peer Support Groups (Virtual Settings) “I wanted to share a group that I follow on Facebook. They're a Longhauler Advocacy group. And there's several people that share on a day-to-day basis of what they go through, resources, any kind of techniques or any kind of medicine or if something's functional that works for them …I kind of felt alone for the longest time.’” (CAT 1) Public Campaigns to Raise Awareness of Long COVID and Other Invisible Illnesses *“Just more outreach of public announcement, just in general … it would be nice for more public awareness to be familiarized*” (CAT 5) *“ So what [the community hospital] does here and there is they'll utilize advertisements …put ads on their buses, on their carpool, share-ride vehicles, Ubers, just the public transports. And you see them everywhere. You'll see like, hey, Alzheimer's. And then also, the other one would be stroke, the signs of stroke, drooping face, and just those little key*, *"Look out for," awareness kind of things. ” (CAT 5)* Educate Family Members, Caregivers, and Clinicians “I would like to see, hopefully … a website online….where it can guide any doctors or PCPs who are unclear about what the process is or who they can reach out to… they even suspect that long COVID might be something that somebody's going through.” (CAT 3) “ I really believe that the education needs to be also not just in the medical people, but your average Joe.” (CAT 1) “It's been a struggle to even get family members to really understand … so I think more awareness would also help patients in the recovery process too.” (CAT 1)
Uncertainty	Improve Communication of Clinical Care Plans “When it comes to the clinical trials and the physician or the specialist … there's just this really big barrier of them being able to communicate that information back and forth.” (CAT 5) Support in Navigating Complex Healthcare and Disability Systems “It's just so hard to even function … let alone getting and doing the paperwork. And that would be such a benefit if there was some type of system or process in place to help folks with that, because I've had to navigate it all on my own.” (CAT 1) Increase Access to Comprehensible Information on New Care Strategies or Research Findings “But [my doctor] had given me a article … it gets very technical … in ways that a person can't comprehend…. So if there can be something that can be a little bit more on a breakdown of [Long COVID] of what it is and what it means and what … could help the patient regain a normal sense or type of living, that would be really helpful.” (CAT 5) Adapt and Accept New Capabilities “[I’m] trying to adapt with what I can do as best to my abilities. I'm trying to do more plans, be a little bit more active” (CAT 2)

“Social media is super important to distribute information because that is … where everybody gets theirs.” (Community Advisory Team Meeting #5)

The potential to reach and influence a wide audience was especially true when the information came from a trusted source, such as a research institution.

“Have your [institutional] credentials … so that people know that you guys are a legitimate research team.” (Community Advisory Team Meeting #5)

Local public health departments and news organizations were also seen as an effective and trusted way of spreading information.

“I was thinking as a potential channel, perhaps … it would be the [local] County Department of Health, and they do a lot of public announcements … They would have a better way of putting out more [information] … if the right people were to go out there and talk to them … Awareness is something that would be beneficial too for the community.” (Community Advisory Team Meeting #4)

### 3.4 Factor 3: uncertainty

Participants described living with ongoing uncertainty about the illness itself and its long-term impact on their lives. This heightened anxiety and complicated decision-making about healthcare and life plans.

#### 3.4.1 Uncertainty about the illness

Questions about the root cause of their symptoms and where to turn for sympathetic physicians and appropriate treatments were largely unanswered. These left patients searching for answers online and through a personal process of trial and error that took months to years.

The uncertainty was compounded by mixed messages from clinicians. One participant described receiving contradictory guidance from two physicians regarding their ability to work. This hindered critical decisions around employment and disability benefits, increasing confusion and stress.

“[The Long Covid specialist] mentioned that I could be on permanent disability if I wanted to, but [the other doctor] says no. He’s not going to sign anything; he’s not going to sign anything. He says I’m too young. Yes, I am young, but he doesn’t know what I’m feeling. Only I know what I feel.” (Interview 8)

In the absence of clear medical guidance, some patients turned to alternative health information sources found online, such as homeopathic and energy-based medicine. These provided seemingly concrete answers and treatments when physicians could not. Others found spirituality to be a grounding force in the face of destabilizing uncertainties. This was especially impactful when symptoms had severely impacted their mental health.

“Besides my medications, I feel that it helps a lot to pray to God, because God, for me, is the one who has been lifting me up since about year ago. I feel that he has helped me overcome many things in my life…” (Interview 8)

Participants reported wanting guidance and support in navigating care and disability systems. Some wondered whether the additional burden of these systemic issues hampered their recovery.

“We’re going into tons of debt. We were … almost homeless on multiple occasions while we were trying to access resources to keep ourself afloat … If there is anything that could be done to help alleviate some of those barriers, I think I’d be very grateful … Maybe some of us could have recovered from the long haul if we didn’t have those extra stressors too.” (Community Advisory Team Meeting #1)

#### 3.4.2 An uncertain life trajectory

Beyond clinical ambiguity, participants expressed a deep uncertainty about their future identities and life plans. The lack of a predictable course of recovery forced many participants to grapple with not knowing whether they would ever feel the same again or be able to return to work and other activities.

These questions about future selfhood and function brought a mix of anxiety and grief. Participants reported feeling suspended in a state of waiting, unsure if they needed to adjust to a “new normal”.

“I’m still really struggling with long COVID … The therapist that I was talking to said I might have to accept that this is my new life. And so that part’s really difficult because I’m hoping that I’ll eventually be who I was again. And I’m not sure that I will. So that’s the part that I’m just trying to cope with, is that this might have to be my new normal.” (Community Advisory Team Meeting #2)

Some participants sought guidance from other Long COVID patients online to explore coping strategies and envision new paths forward, but uncertainty lingered.

“They’re a Longhauler Advocacy group. And there’s several people that share on a day-to-day basis of what they go through, resources, any kind of techniques or any kind of medicine or if something’s functional that works for them as some kind of pro tips on how to get across what we’re going through.” (Community Advisory Team Meeting #1)

## 4 Discussion

This study advances our understanding of the multi-faceted aspects impacting psychosocial well-being among Long COVID patient populations, and the potential avenues for improvement. While considerable research has established the physical and cognitive symptoms of Long COVID, such as post-exertional malaise and brain fog, patient perspectives on how these translate into lived experiences of disrupted psychosocial well-being, and what should be done to support psychosocial well-being, remain under-examined. Our results highlight the intersecting impacts of *identity disruption*, *isolation*, and *uncertainty*. Notably, these factors may not be standalone but rather amplify or otherwise affect one another. For instance, uncertainty may exacerbate isolation, or identity disruption may fuel uncertainty. Nevertheless, these three overarching factors can be understood and potentially addressed through the lens of established well-being frameworks, such as Stanford’s WELL model and Maslow’s Hierarchy of Needs (32–35).

With interventions tailored to Long COVID still in development, current practices are largely modeled after those used to treat psychosocial well-being for other similar chronic conditions, such as chronic fatigue syndrome (CFS), fibromyalgia, chronic pain syndromes, post ICU syndrome, and autoimmune diseases (4). Cognitive behavioral therapy (CBT) is one such practice that has shown measurable success in treating individuals with ME/CFS and fibromyalgia, leading to improvements in mood, coping strategies, and overall quality of life (40, 41). Many of the participant recommendations that arose in this study parallel interventions that have been applied to other chronic conditions.

### 4.1 Identity disruption: supporting adaptation, self-compassion, and changing roles

One of the most prominent insights across interviews and focus group discussions was the harm of a disrupted personal identity. Many participants expressed a painful sense of disconnection from their former selves, describing this as a feeling of not being who they used to be. Such experiences are frequently reported with other chronic illnesses such as ME/CFS and fibromyalgia (42, 43). This identity disruption reported among Long COVID patients relates to the *sense of self* and *sense of purpose and personal growth* WELL domains, which include elements of physical and cognitive functioning, self-compassion, values, passions, and the development of new skills and perspectives (33, 34).

The connection between identity disruption and related WELL domains provides a potential basis for future interventions. Our participants suggested cultivating gratitude and positivity as strategies for adapting to changes in identity and capability (**Table 3**). Building on this patient-generated recommendation, existing literature suggests that cultivating self-compassion-based approaches, such as loving-kindness meditation, may help reduce distress associated with identity disruption, support positive adaptation to changes in identity, and restore or establish a coherent and affirming sense of self (44, 45). Such approaches may be relevant for individuals with Long COVID trying to bridge the gap between who they were before their illness and who they are now. Importantly, self-compassion-based interventions represent a literature-informed extension of participants’ broader recommendations around acceptance, gratitude, and adaptation rather than an intervention directly proposed by participants.

### 4.2 Isolation: reconnecting through peer support networks and campaigns promoting awareness of Long COVID

A second key theme of experiences with Long COVID was isolation, which aligns with the *loneliness* and *social connectedness* WELL domains (33, 34). Across our patient narratives, participants identified Long COVID support groups as a potential strategy for addressing isolation by providing opportunities to connect with others who share similar experiences (**Table 2**). This is consistent with literature on chronic illness communities, where peer interactions have been shown to build social connection and may reduce isolation and loneliness (46). This participant-generated strategy is also supported by emerging Long COVID-specific literature, both observational and interventional, suggesting that peer support may foster validation, social connection, information sharing, and a sense of community (47, 48). One eight-week Long COVID-specific peer support intervention also found a small-to-moderate decrease in depression severity following participation (49). Although evidence remains limited, the findings of the present study support further evaluation of structured peer-support interventions for Long COVID.

Participants also called for an awareness campaign tailored to Long COVID that could help normalize the condition, promote understanding of invisible symptoms, and encourage supportive behaviors from family, community members, and clinicians (**Table 3**). Evidence from mental health stigma campaigns suggests that population-level awareness efforts can effectively shift public attitudes and reduce stigma (50). Future Long COVID awareness interventions could implement broad communication strategies and evaluate subsequent awareness, and whether this translates into greater perceived validation and social support by patients.

### 4.3 Uncertainty: material, logistical, and personal support strategies

Uncertainty was a recurring theme across participant narratives, aligning with the *resilience* domain of the Stanford WELL framework and the *physiological* and *safety needs* domains of Maslow’s Hierarchy of Needs (32–34). Participants identified strategies for reducing this uncertainty that centered on greater practical support, assistance navigating complex systems, and cultivating personal practices to manage remaining uncertainty.

Participants described difficulty accomplishing daily tasks including grocery shopping and traveling to medical appointments due to fatigue, breathing issues, brain fog, and other cognitive symptoms (**Table 2**). Job loss further contributed to financial insecurity, housing instability, and food scarcity (**Table 2**). In response, participants emphasized the potential value of greater material support, including assistance with daily needs (**Table 3**). These patient-identified needs and strategies may be addressed through existing interventions that have been applied to other populations, such as financial assistance (51), grants, food delivery services (52), and part-time caregivers, which may help reduce the risks associated with job loss and decreased physical abilities. Participants described how even knowing support services existed could ease concerns regarding what might happen if they needed assistance, even if they did not end up using the services (**Table 3**).

Managing healthcare logistics added to the burden of Long COVID. Participants described the toll of coordinating specialty care while navigating complex insurance and disability benefit systems, often reporting that they were not sure what steps to take next. Participants suggested that case managers or similar navigational support could help guide Long COVID patients through these systems, thereby reducing uncertainty surrounding the care process (**Table 3**).

Existing evidence supports this participant-suggested approach. A 2015 study of case management for patients with chronic diseases, in which nurse case managers helped patients develop personalized service plans and coordinate with healthcare providers, found that patients reported greater clarity on their healthcare process, improved communication and coordination regarding their care, and felt that their needs were heard and appropriately managed (53). While this case management model was developed in a general chronic disease population in Canada, which has universal healthcare, it could potentially be adapted for Long COVID in the U.S. by accounting for differences in healthcare costs and accessibility, as well as illness-specific barriers such as stigma. These findings are bolstered by a 2018 systematic review of case management in chronic illness populations, which found increases in healthcare access and emotional support (54). Together, these findings provide a rationale for evaluating whether case management models could address the navigational needs and uncertainty identified by participants with Long COVID.

Participants emphasized the value of developing personal practices to make it through difficult days and weeks, with several discussing efforts to maintain a positive outlook or accept their current situation (**Table 3**). This participant-suggested approach to managing uncertainty experiences aligns with the concept of *resilience*, a key domain of well-being that involves adapting to hardships and uncertainties (55). As with the other well-being domains, resilience is not pre-determined and can be cultivated to shape how individuals navigate future challenges (56). One such intervention demonstrated to strengthen resilience involves practicing mindfulness (57). This is a meditative practice that involves focusing on present-moment internal or external experiences with the aim of accepting what is felt (57). Although the specific term mindfulness was not itself identified by participants as a recommendation, it closely relates to the adaptation and acceptance that participants described as important.

### 4.4 Limitations

The small, convenience-based sample (*n* = 19) from Northern California may limit generalizability, though in-depth exploration allowed for rich insights into the lived experiences of those with Long COVID. Participants were also recruited from clinics and community health fairs, limiting representation to those who were seeking care or well enough to attend. This introduces selection bias that may have shaped the relative prominence of identity disruption, isolation, and uncertainty in our findings, as individuals who are less connected to healthcare or community resources may experience these factors differently or encounter additional psychosocial challenges not captured in our sample. This approach was pragmatic and helped identify participants who were able to provide detailed, experience-based accounts. Additionally, incomplete demographic reporting limited our ability to characterize the sociodemographic composition of the sample and examine how participant characteristics may have shaped the experiences represented in the findings. Since this study represents a secondary analysis of data collected for a broader research effort, data collection was not guided by thematic saturation.

Another limitation was variability in attendance across CAT sessions. Because each session addressed a different topic, lower attendance in some sessions may have reduced longitudinal consistency by resulting in different subsets of participants contributing to different topics. Additionally, CAT sessions were conducted only in English due to facilitation constraints. Although Spanish-speaking participants were included in individual interviews, language-related differences across data collection modalities may have influenced the perspectives captured. The acceptability and accessibility of certain proposed interventions, such as mindfulness-based practices and virtual peer support, may also vary across cultural and linguistic contexts. Finally, while mitigation strategies presented in this article are built from both participant suggestions and applicable interventions in existing literature, they have not been comprehensively reviewed by study participants. This highlights the need for future participant involvement in collaboratively designed studies.

## 5 Conclusion

Effectively treating and supporting individuals with Long COVID requires consideration of their broader state of psychosocial well-being. Key factors influencing this well-being include *identity disruption*, *isolation*, and *uncertainty*, each of which represents a potential target for supportive interventions. Building on our findings and participant-generated recommendations, patient-informed approaches to addressing psychosocial well-being for Long COVID could include the following: self-compassion training to address identity disruption, structured peer support networks and public awareness campaigns to reduce isolation, and individualized case management paired with mindfulness training to improve access to resources and manage uncertainty. While no single intervention will fully eliminate the invisible burden of Long COVID, these patient-informed strategies provide promising starting points for future research aimed at improving care and quality of life for individuals living with this disease.

## Data Availability

To protect the anonymity of our participants, the qualitative data supporting the conclusions of this article are not publicly available in full but are available from the corresponding author upon reasonable request.

## Supplementary material

Supplementary Table 1 Factors Influencing Psychosocial Well-being, Extended Quotes.
