# Supplementary material for: Supporting the psychosocial well-being of patients with Long COVID: patient-informed strategies from a qualitative study
Source: Front Psychiatry. 2026 Sep 23;17:1837359. doi: 10.3389/fpsyt.2026.1837359 (PMC13642708; doi:10.3389/fpsyt.2026.1837359)
Supplement: Supplementary file 1 [file Supplementaryfile1.docx]

**Supplementary Material: Semi-Structured Interview Guide**

Introduction

1. Tell me the story of your experience with Long COVID.

Patient Identity and Self-Perception

1. Can you tell me how living with Long COVID has affected how you see yourself or your role in your life – such as in your family, work, or community?
2. What have you found to be most helpful in navigating difficult experiences with Long COVID?

COVID-19 Care

1. When did you decide to seek care for Long COVID? Did you know that’s what you had?
2. What has it been like for you to talk with doctors or healthcare providers about your experience with Long COVID?
3. What could be improved?

Referral Experience

1. What has been your experience in being referred to different doctors – have you been referred to or seen any specialists?

Needs, Support, and Caregivers

1. Aside from medical care, what else has been helpful in adjusting to having Long COVID? Have you wanted support with other things? (finances, social support, caregiving)?
2. What is your experience with caregivers? This could be anyone that has helped you (a parent, a friend, a volunteer, etc.).

Community Engagement and Education

1. Where do you go to find information on Long COVID? For health information generally?
2. What education do you still need or want about Long COVID? What would have helped you during your diagnosis?
3. How would you describe your community’s awareness of Long COVID? (family, neighborhood, state, faith organization, etc.)
4. What do you think would be the best way to raise awareness and share information about Long COVID, and health more generally, in your community?

Experience with Bias in Medicine

1. Can you tell me what bias means to you?
2. Can you tell me about a time when you experienced bias when you got medical care?
