# Supplementary material for: Supporting the psychosocial well-being of patients with Long COVID: patient-informed strategies from a qualitative study
Source: Front Psychiatry. 2026 Sep 23;17:1837359. doi: 10.3389/fpsyt.2026.1837359 (PMC13642708; doi:10.3389/fpsyt.2026.1837359)
Supplement: Supplementary file 2 [file Table1.docx]

**Supplementary Table 1: Factors Influencing Psychosocial Well-being, Extended Quotes**

| **Factor** | **Exemplar Quotes** |
| --- | --- |
| **Disruption of Personal Identity** | - *“I used to be a gym-going person, very outgoing before COVID. I've significantly gained a lot of weight due to my inability of being active with fitness and such.” (CAT 3)* - *“Have you ever woke up and just wanted to say, 'All right. We're going to give it a go no matter what…I mean, give it your 100% to match your life as it once stood before getting hit with long COVID.' I have days that my mind is determined. 'I am going to ignore everything that ails me,' so I commit to the day. 7:00 AM, I go about handling yard work, light gardening, move on to running errands, grocery shopping, kids' school meetings, helping elder families to get doctors' appointments, assist another family member with Parkinson's with care at his home, commute, drive home, prepare dinner, handle some laundry. By 6:00 PM, I already cannot ignore my body screaming to shut down. Energy is beyond spent. Muscular pain is intense. Breathing is labored. Sitting and standing is a massive concern because you feel like you will collapse. Legs and arms are heavy. You cannot push through it anymore. You call it a day. Laying down, your mind is still racing that there is unfinished tasks, but your body flat out says no. The next day, you are riddled with immense body pain. And moving around just to do the simplest of tasks is just too much. You already know in the back of your mind that you have to conserve your energy just to get through the day. So you tread lightly. Well, folks, that's me.”(CAT 2)* - *“It's just been a bit different with my concentration. Most of the time, I have to sit in front to be able to fully understand what the professor is saying without getting lost or tired. It also, in a way, affects my studying. Either I have to be straight on caffeine to be able to concentrate and stay awake in a way because even if I eat right, even if I have enough vitamins or have three meals, I still feel pain and fatigue and a bit of brain fog.” (Interview 13)* - *“Adulting used to be really easy for me. And I just feel like a child at this point. It's really hard for me to do pretty much anything. I used to be super organized.”(CAT 3)* - *“I had always been a top salesperson at the company for five years, but I just couldn't keep up anymore… I would go to bed and hope that I never wake up again because that’s just how exhausted my body was.”(CAT 3)* - *“The hardest thing for me has been that, before I got COVID, I was a very active woman…I didn’t depend on anyone… and now I can’t fend for myself because I’m too afraid to drive, to go out and everything.”(Interview 8)* - *“My biggest issues are being able to articulate myself like I once was.”(CAT 2)* - *“At work, when I'm tired, I try to just keep moving more so I don't go to sleep because I'm like, "I don't want to get fired because I'm sleeping on this job.” (Interview 7)* - *“I could not get close to people because I was afraid of them. Now I can be around people, socialize a little more, but before, no. I didn’t want to get close to anyone. And I didn’t want anyone to get close to me either. I go to the store by myself… I know and hope that little by little I am going to improve my life. It has been very difficult, but nothing is impossible.”(Interview 8)* |
| **Experiences of Isolation** | - *“ I go through these immense crashes where I literally cannot talk to anybody. I don't want to—I can't communicate through text, through email. I mean, this is quite ridiculous, but just to share how many texts are on my cell phone that I have not even answered are 347.” (CAT 2)* - *“Because when we're going through these slumps with no energy, it's almost hard to do really anything and have conversations with people. So that has prevented me a lot of the time from having a lot of progress with getting through it all just because it takes so much time and energy.” (CAT 1)* - *“I’ve always been social, and I have become a complete recluse because I don’t know how to explain what I’m going through to people. And I tried once to one of my very best friends, and she didn’t understand.…I just realized unless somebody’s going through it, it’s just not really worth my energy to try and explain it to somebody.… And I just have found too, that unless people are going through it, which I don't know anybody in my own friend of circles that I've grown up with, it's just easier for me to just disengage.”(CAT 2)* - *“I've dealt with friends and family saying, ‘Get over it already… You are fine. Nothing is wrong with you. You just got lazy.’ No one will ever know or truly realize that both mental and physical stressors are your true enemy now.” (CAT 2)* - *“And I've become a hermit, pretty much - I'm getting a little better - because I don't trust other people with their germs…So it really limits me socially, to be around people…And I know it bothers my family because they want me to go places and do things. Let's say they want to go to a concert. I'm not going to go because, I mean, I can't control it.” (Interview 13)* - *“Most of the things I have experienced were headaches and fatigue. And I was no fun to be around because I was always in pain. And I've gotten a lot of complaints as in, "Oh. Why don't you just go get it checked out? Why are you always so weak? Why are you always in pain? Why are you always so sick?” (Interview 12)* - *“So little by little, I'm just trying to advocate for myself and also trying to find any other resources. I know there's a couple of groups also on Facebook, on social media. There's a long COVID group that's called COVID-19 Long Hauler Advocacy Project. And they have several people who actually talk to senators…to see if we can get better recognition.” (CAT 1)* |
| **Uncertainty** | - *“Why do some people heal with COVID and some people don't? I just don't know… I started doing craniosacral. I was starting counterstrain, acupuncture… What else have I done? Massage. I soak my feet in something called fulvic minerals.” (Interview 2)* - *“I'm still really struggling with long COVID… The therapist I was talking to said I might have to accept that this is my new life. That part’s really difficult because I’m hoping that I’ll eventually be who I was again. And I’m not sure that I will.”(CAT 2)* - *“[The Long COVID specialist] mentioned that I could be on permanent disability if I wanted to, but [the other doctor] says no. He's not going to sign anything; he's not going to sign anything. He says I'm too young. Yes, I am young, but he doesn't know what I’m feeling. Only I know what I feel." (Interview 8)* - *“But I have been investigating, as we should not just rely on medicine. I was not aware of so many things. In my case, besides my medications, I feel that it helps a lot to pray to God, because God, for me, is the one who has been lifting me up since about year ago. I feel that he has helped me overcome many things in my life, things that… when I felt so desperate because of my symptoms, sometimes I wanted to commit suicide. I'm going to put it this way. I wanted to kill myself and I didn't want to live anymore. Even last year, in December, when I had mental lapses, I felt in a great darkness in me and I thought, "I have felt tired since this disease came into my life." I never complain about my illnesses.”(Interview 8)* - *“TikTok is very informational… if you get on the right thread, the right algorithm of health issues or homeopathic things, like Barbara O'Neill…she is from Australia, and she's a homeopathic doctor. And she does seminars all across the United States. Mostly East Coast. But she's Christian, so she bases her teachings off of the Bible, and talks about how wheat is not the wheat that God intended for us to eat.”(Interview 4)* - *“I've bought so many different types of multivitamins because over time, I've been feeling very fatigued. I'm always sick, always have headaches. I thought it was migraines, but migraines feel different. And I have a cabinet full of just, multivitamins. And even halfway through the bottle, I had not seen any healthier or better results from it. So I would just give up and then I'd get sick again. And I'd say, "Okay, maybe different vitamins." And I have a cabinet full of supplements, so for immunity boosters, vitamin C tablets. I buy a lot of oranges, buy a lot of spinach for iron, magnesium, fiber, lots of avocados, healthy carbs, potatoes, rice, lots of veggies. But yet still with that, I get sick very, very easily, fatigue very, very easily.” (Interview 12)* |
